# Demineralization Depth Using QLF and a Novel Image Processing Software

**DOI:** 10.1155/2010/958264

**Published:** 2010-04-28

**Authors:** Jun Wu, Zachary R. Donly, Kevin J. Donly, Steven Hackmyer

**Affiliations:** ^1^Dental Branch, University of Texas Health Science Center at Houston, 6516 John Freeman Boulevard, Houston, TX 77030-3402, USA; ^2^Department of Pediatric Dentistry, Dental School, University of Texas Health Science Center at San Antonio, 7703 Floyd Curl Drive, San Antonio, TX 78229-3900, USA

## Abstract

Quantitative Light-Induced fluorescence (QLF) has been widely used to detect tooth demineralization indicated by fluorescence loss with respect to surrounding sound enamel. The correlation between fluorescence loss and demineralization depth is not fully understood. The purpose of this project was to study this correlation to estimate demineralization depth. Extracted teeth were collected. Artificial caries-like lesions were created and imaged with QLF. Novel image processing software was developed to measure the largest percent of fluorescence loss in the region of interest. All teeth were then sectioned and imaged by polarized light microscopy. The largest depth of demineralization was measured by NIH ImageJ software. The statistical linear regression method was applied to analyze these data. The linear regression model was *Y* = 0.32*X* + 0.17, where *X* was the percent loss of fluorescence and *Y* was the depth of demineralization. The correlation coefficient was 0.9696. The two-tailed t-test for coefficient was 7.93, indicating the *P*-value = .0014. The *F* test for the entire model was 62.86, which shows the *P*-value = .0013. The results indicated statistically significant linear correlation between the percent loss of fluorescence and depth of the enamel demineralization.

## 1. Introduction

Dental caries is the most prevalent chronic disease in children. Although there is evidence that the prevalence of dental caries has significantly decreased over the past 20 years [1, 2], dental and oral diseases continue to plague children, especially young children. According to reports of the Centers for Disease Control and Prevention (CDC) comparing National Health and Nutrition Examination Surveys (NHANES), about 28% of preschool children experienced tooth decay between 1994 and 2004 [3, 4]. 

The development of dental caries is a dynamic disease process, especially for early lesions, which have repeated demineralization and remineralization cycles before being clinically detected. Demineralization occurs from acidic substrate or carbohydrate fermentation by acidogenic microorganisms, causing a subsurface enamel lesion to form. The continuation of demineralization leads to cavitation on the enamel surface. Restorative dentistry is often required at this stage. Untreated dental decay will cause pain and possible premature tooth loss which can be harmful to the permanent dentition and can cause tooth crowding problems, speech disorders, compromised chewing, delayed growth and development, and high treatment costs [5–7]. The natural repair response to demineralization is remineralization, which incorporates minerals from saliva into the demineralized lesion. Due to the ubiquitous use of fluoride, the progression of enamel caries becomes slower. It is likely that many incipient lesions could be arrested before they become clinically detectable [8, 9]. Fluoride improves saliva remineralization effects and forms an acid-resistant fluorapatite-rich surface of enamel. The fluoride in plaque also interferes with the bacterial metabolism with a subsequent decrease acid production [10].

The slow progression of enamel caries offers the opportunity for dental professionals to diagnosis and manage caries before there is irreversible destruction of the tooth. “With respect to dentinal caries, the diagnosis of the disease and the detection of early lesions should be regarded as cornerstones of cost-effective dental health care delivery and quality of care.” [11] However, there exist large variations in caries diagnosis and treatment decisions due to the lack of reliable methods to analyze the extent of the subsurface decay [12–18]. 

Traditional diagnostic methods, such as visual inspection, aided by radiography with or without tactile sensation, appear to have low sensitivity and high specificity for caries detection. The translucency, color, hardness, and radiographic interpretation are factors that lead to a dichotomous decision (either absence or presence of caries). Although they are simple, quick, and cost-effective, the methods have considerable limitations. The earliest lesions are detected at the white spot stage. In addition, the demineralization and remineralization processes are not quantifiable to be monitored with the current diagnostic techniques routinely utilized today. The use of an explorer to forcefully probe tooth surfaces may cause damage to newly erupted teeth or create cavitation at superficial lesion sites [19–23]. Radiographs are the most widely used diagnostic technique in conjunction with visible examination, but is limited to interproximal enamel caries detection. 

A new visual method, the International Caries Detection and Assessment System (ICDAS), was developed to provide an international system for recording detected caries and comparing data collected in different locations at different points of time [24, 25]. The visual examination is carried out on clean, plaque-free teeth, aided by a ball-ended explorer to check surface contour, minor cavitation or sealant.

In recent years, many new diagnostic systems have been developed based on the measurement of different physical signals, such as visible light, laser light, electronic current, and ultrasound [26]. Fiber-optic transilumination (FOTI) is an advanced visual inspection technique based on light scattering properties in enamel. When the light is placed on the buccal or lingual side of tooth, the light is scattered in the enamel to result in a relatively darker region in demineralized areas, compared to sound tissue. This contrast is used to detect dental lesions, especially for inter-proximal carious lesions, and has shown low to good sensitivity and good specificity [27–30]. However, this technique still cannot be quantified or well documented in longitudinal studies. The digitized fiber optic transillumination (DI-FOTI) is an improved FOTI technique to collect transmitted images displayed on a computer monitor for evaluation, but the evaluation is still undertaken by the examiner's subjective visualization [31–35]. DIAGNOdent (KaVo, Biberach, Germany) is a different technology from FOTI to detect carious lesions, based on the difference in fluorescence between sound and demineralized enamel. The device uses a small laser system to produce an excitation wavelength of 655 nm, which is transmitted through optic fiber to a hand-held probe. The excitation light from the probe tip is absorbed by both organic and inorganic tooth substances. The emitted infrared fluorescence is collected by the probe tip and quantified to be displayed on an LCD panel. This technique has high sensitivity and specificity, especially for carious lesions on occlusal surfaces [36–39]. However, the device supplies the information as an arbitrary value, and has to be calibrated frequently for longitudinal comparisons. The probe must also be rotated in all directions to detect the highest reading, which is very technique sensitive.

Another dental diagnostic tool for detection of early carious lesions is quantitative light-induced fluorescence (QLF), which is based on auto-fluorescence of teeth. When the teeth are illuminated with high intensity blue light, the resultant auto-fluorescence of enamel is detected by an intraoral camera which produces a fluorescent image. The emitted fluorescence has a direct relationship with the mineral content of the enamel [40–44]. Thus, the intensity of the tooth image at a demineralized area is darker than the sound area. The software of QLF systems can process the image to provide user quantitative parameters such as lesion area, lesion depth, and lesion volume. These parameters can detect and differentiate the lesions at very early stages, and make the QLF system more sensitive to changes of caries over time. The image can be stored for longitudinal study and be used as patient motivators in a preventative practice [45–47].

QLF has been widely used as a quantification system for assessing early demineralization or remineralization of human enamel by thoroughly investigating the correlation between fluorescence loss and the status of mineralization under various treatments. In these studies, the changes of lesion area, depth, and volume are expressed as changes of fluorescence in the region of interest. In order to detect the stage of early demineralization, the absolute lesion depth needs to be quantified. Some studies have found a strong correlation between the changes in lesion and fluorescence with a current gold standard methodology-Transverse Micro-Radiography (TMR) [41, 48–50]. However, these validation tests prepared samples from teeth that were cut and ground to flat surface enamel, and the fluorescence loss is based on the difference of average fluorescence on acid treated and control areas. 

In our study, we use teeth with natural surface curvature, without cutting or grinding the enamel surface flat, then validate the interpolation algorithm of QLF technology to estimate the changes of fluorescence. The purpose of this in vitro project was to simulate the clinical intraoral situation, and investigate the correlation between the fluorescence loss and demineralization depth, so that the absolute lesion depth could be estimated to evaluate the stage of early demineralization. In addition, according to the QLF image processing algorithm, there are random errors in reconstruction of sound values, and the measured maximum fluorescence loss from a single pixel is extremely sensitive to random noise [51]. We improved the QLF image processing algorithm and implemented it into a novel software, which produces more reliable results.

## 2. Methods and Materials

Six extracted permanent molars were obtained from different individuals with various ages and various exposures to fluoride histories. The teeth were stored in 0.1 percent thymol solution. The teeth were examined with a light microscope at 10 times magnification to see that no white spot lesions or enamel imperfections were present. The teeth were coated with an acid-resistant varnish, leaving a 1 × 5 mm window of enamel exposed. The teeth were placed in an artificial caries solution (2.2 mM Ca^+2^, 2.2 mM PO_4_^−3^, 50 um acetic acid) for four days to produce incipient demineralized enamel lesions [52]. 

The acid-resistant varnish was then removed carefully with acetone and the teeth were placed in deionized distilled water. Teeth were taken from the water, air dried, and a QLF image was obtained (Inspektor, Amsterdam, Netherlands).

The teeth were then cut longitudinally with a hard tissue microtome (Silverstone-Taylor; Scientific Fabrications, Lafayette, CO, USA), to obtain 100 um sections. These sections were photographed, using a polarized light microscope (Olympus; Model BX60FS, Olympus Optical Co., LTD., Tokyo, Japan) in an imbibition media of water, representing greater than one percent pore volume [53]. The lesion depths on these sections were measured with a computerized imaging system (NIH ImageJ software, http://rsbweb.nih.gov/ij/). 

### 2.1. QLF Image Analysis

**Figure 1:**
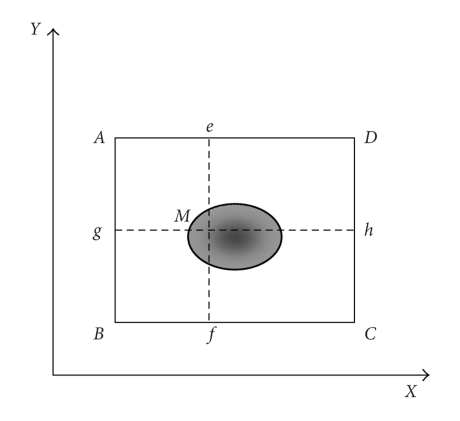
Interpolation description. The dark area represents lesion and others represent sound tooth structure. Thus, lines *A* *B*, *B* *C*, *C* *D*, and *D* *A* were seated on sound tooth structure and were used for interpolation.

The principle of QLF software in analyzing the loss of fluorescence is “a two-step, two-dimensional, linear interpolation of the fluorescence radiance values at the sound edges of the lesion area” [51]. For example, to calculate the lesion at point *M* (intercross of line *e* *f* and line *g* *h*), the first step calculates the linear interpolation value *L* *m*(*x*) parallel to the *X*-axis (Figure 1) as

(1) $$Lm\left(x\right)=Lg+\left(Lh\text{-}Lg\right)*\frac{\left(Xm\text{-}Xg\right)}{\left(Xh\text{-}Xg\right)},$$

where *L* *g* and Lh are intensity values at points *g* and *h* in the QLF image. The (*X* *m*-*X* *g*) is length of line *M* *g*, and (*X* *h*-*X* *g*) is length of line *gh*.

The second step begins to calculate interpolation values *L* *e*(*i*) and *L* *f*(*i*) at points *e* and *f* using intensity values at *A*, *D*, and *B*, *C* as step 1. The intensity differences between real intensity (*L* *e*, *L* *f*) and interpolation intensity (*L* *e*(*i*), *L* *f*(*i*)) at points *e* and *f* are calculated as

(2) ΔLe=Le−Le(i)  ,  ΔLf=Lf−Lf(i).

Then, the linear interpolation parallel to *y* axis is calculated as

(3) $$Lm\left(y\right)=\Delta Le\;+\;\left(\Delta Lf\text{-}\Delta Le\right)*\frac{\left(Ym\text{-}Ye\right)}{\left(Yf\text{-}Ye\right)},$$

where (*Y* *m*-*Y* *e*) is length of line *M* *e* and (*Y* *f*-*Y* *e*) is length of line *fe*.

Finally, the desired interpolation value at point *M* is *L* *m*(*i*) = *L* *m*(*x*) + *L* *m*(*y*).

The loss of fluorescence at point M is Δ*L* *m* = *L* *m*(*i*) − *L* *m*, where *L* *m* is the lesion intensity at *M* in the QLF image. By comparing Δ*L* at each lesion pixel, the maximum value of fluorescence loss is defined as Δ*L*_*max*_.

However, there are significant errors associated with Δ*L*_max _ measurement. Δ*L*_max _ is taken from a single pixel value, which could be dramatically affected by random noise such as a hot or cold spot. Another potential error is a reconstruction error, which depends on the accuracy of each pixel value (fluorescence on sound tooth structure) on lines of *A* *B*, *B* *C*, *C* *D*, and *D* *A*. The random noise at these lines also can significantly affect interpolation values at lesion area. In addition, the rectangle may not fit appropriately for irregular lesion shapes. The edges may be far away from or interfere with lesion area.

To obtain precise measurements, we modified the interpolation algorithm and developed an image processing software. Instead of a rectangle, our software supports users to draw a polygon so that all edges can be on sound tooth structure and closely adapted to lesion shape. To reduce the errors for interpolation, the intensity values of each pixel on the polygon edges were recalculated as mean values of a 3 × 3 pixel area. In addition, to reduce the computational error of Δ*L*_max _, the averages of 3 × 3 pixels of the Δ*L* value at the same site as that of Δ*L*_max _ were compared. If there was significant difference between the mean value and Δ*L*_max _, the large error was associated with Δ*L*_max _. Then the second largest Δ*L* was selected for evaluation and the mean value of Δ*L* in a 3 × 3 pixle area represented the largest loss of fluorescence. This data was used to build a statistical model to estimate lesion depth.

### 2.2. Data Analysis

Microsoft Excel was used to analyze the data. The linear regression model was generated and an *R*-squared value was calculated.

## 3. Results

**Figure 2:**
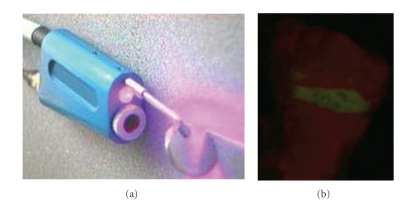
Fluorescent tooth image (b) collected by Quantitative Light-induced fluorescence [QLF] (a).

**Figure 3:**
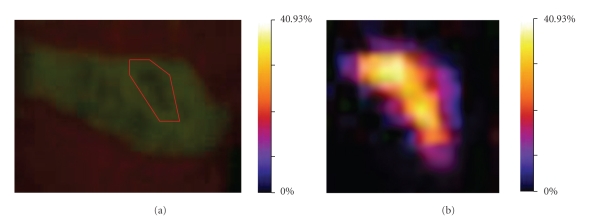
QLF images were analyzed by a novel image processing software. The region of interest [ROI] is shown as the red polygon (a). The resulting image of ROI [magnified ×5] after processing is colored according to the Fire Look-Up Table [LUT] (b).

**Figure 4:**
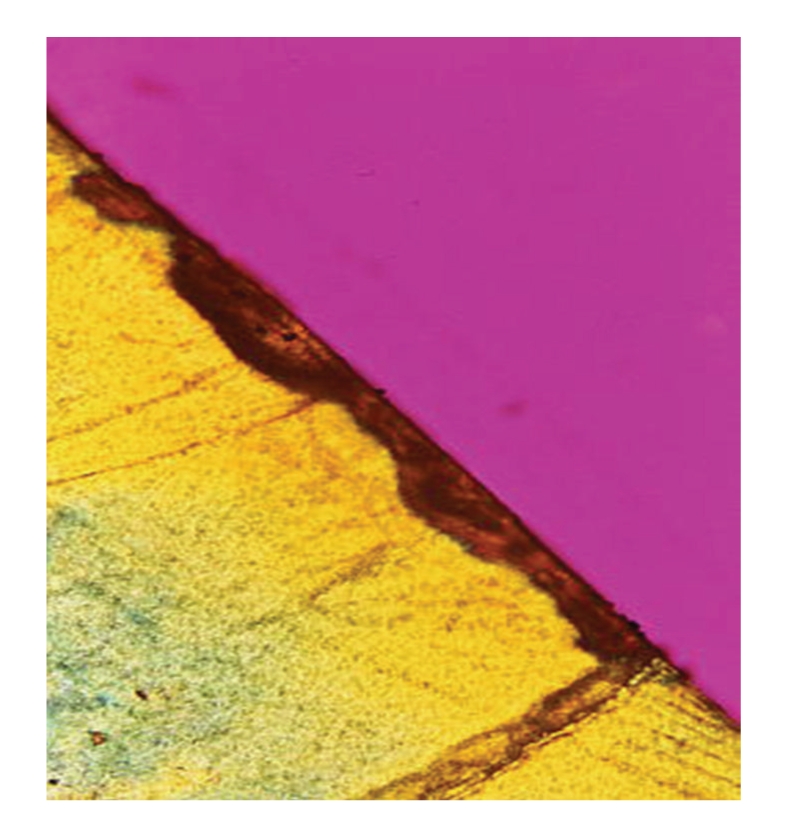
The largest caries depth [*d*] is measured.

The images collected by QLF (Figure 2) were processed. The region of interest (ROI) was selected, and then the interpolation methods were applied to calculate the percentage loss of fluorescence. The resulting image was displayed by Fire lookup table (LUT), and the associated color scale is shown (Figure 3). The teeth were sectioned and imaged by polarized light microscopy (Figure 4). The deepest demineralization depth was measured as distance “*d*” by imageJ software. The largest depth data of demineralization and the most percentage loss of fluorescence are listed in Table 1. 

**Figure 5:**
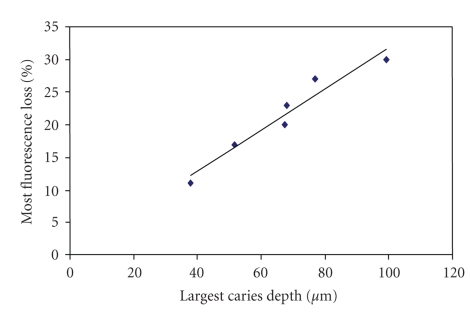
Linear regression analysis graph.

The statistical linear regression was applied to these data (Figure 5). The correlation coefficient was 0.9696. The two-tailed *t*-test for coefficient was 7.93, indicating the *P*-value = .0014. The F test for the entire model was 62.86, which shows the *P*-value = .0013. Thus, results show significant linear correlation between the demineralization depth (*X*) and fluorescence loss (*Y*) as *Y* = 0.32*X* + 0.17.

## 4. Discussion

The results indicated statistically significant linear correlation between the percent loss of fluorescence and depth of the enamel demineralization. This would facilitate in-vivo measurement of demineralization using the noninvasive QLF technique, based on this linear model. This method provides dentists with critical information about the depth of demineralization when treatment planning dental care for patients. 

Although extrapolating the findings for in vivo application may have potential, careful evaluation of how the oral environment, especially the moisture component and the angulation of teeth, may affect the QLF reading should be examined. 

Compared to in vitro experimental conditions, in vivo application of QLF has certain difficulties, including access to lesions on occlusal and interproximal surfaces, measurement reliability, the effect of incorporation staining in lesions or sound surface, moisture in the oral environment, and angulations of light source. 

The occlusal surface has complicated anatomic structures so that a complex light scattering pattern is generated, which may result in poor contrast between demineralization and sound tissue. A current approach to detect occlusal lesions is DIAGNOdent, where excited 655 nm wavelength light from the probe tip and emitted infrared fluorescence is collected and quantified as an arbitrary value to display on an LCD panel. However, the device needs to be calibrated for every usage, and is technically sensitive to angulation of the probe tip and occlusal staining. 

Compared to the bulk of sound enamel structure on facial and lingual surfaces, the interproximal surface has its own complicated light-scattering properties, and the light scattering can also interfere with adjacent teeth. The QLF approach to detect interproximal lesions needs to be evaluated with other techniques such as FOTI or DI-FOTI using transmitted light. 

Surface staining may cause technique difficulties to differentiate the lesion from sound tooth structure [54, 55]. The conventional professional prophylaxis is recommended before QLF application. Intraoral moisture may have high impact on QLF results. In vitro experiments apply air dry to teeth before QLF application. In dry teeth, the scattering of light is increased since the refractive index of dry enamel crystals is much greater than that of wet enamel crystals. Thus, in order to standardize the in vivo test, the drying time must be consistent [56]. In addition, the surface smoothness and curvature, and the angulation of the light source to tooth surfaces need to be evaluated by in vivo studies. 

In this study, we modified the interpolation algorithms using the mean value of ROI (Region of Interest) border to interpolate the inside lesion area. The fluorescence loss at each pixel in the lesion is recalculated as the mean values of 3 × 3 pixel areas to avoid possible cold spots, which are created by system random error. In our study, we did find certain cold spots with significant fluorescence loss compared to its adjacent pixels in the QLF image (data not shown). These modifications minimize the system error so that the data is more reliable. 

Early intervention with remineralizing agents could be evaluated for effectiveness, QLF examination at recall appointments allowing the clinician to see improvement or advancement in lesion depth.

## Figures and Tables

**Table 1:** List of data for building statistical linear regression model.

Largest Caries Depth (*μ*m)	37.8	51.6	67.6	68	77	99.2
Most Fluo. Loss (%)	11	17	20	23	27	30
